# Real-Time Parallel Artificial Membrane Permeability Assay Based on Supramolecular Fluorescent Artificial Receptors

**DOI:** 10.3389/fchem.2020.597927

**Published:** 2020-11-03

**Authors:** Suhang He, Anxhela Zhiti, Andrea Barba-Bon, Andreas Hennig, Werner M. Nau

**Affiliations:** Department of Life Sciences and Chemistry, Jacobs University Bremen, Bremen, Germany

**Keywords:** drug discovery, permeability, fluorescence, cucurbituril, passive diffusion

## Abstract

Parallel artificial membrane permeability assay (PAMPA) is a screening tool for the evaluation of drug permeability across various biological membrane systems in a microplate format. In PAMPA, a drug candidate is allowed to pass through the lipid layer of a particular well during an incubation period of, typically, 10–16 h. In a second step, the samples of each well are transferred to a UV-Vis–compatible microplate and optically measured (applicable only to analytes with sufficient absorbance) or sampled by mass-spectrometric analysis. The required incubation period, sample transfer, and detection methods jointly limit the scalability of PAMPA to high-throughput screening format. We introduce a modification of the PAMPA method that allows direct fluorescence detection, without sample transfer, in real time (RT-PAMPA). The method employs the use of a fluorescent artificial receptor (FAR), composed of a macrocycle in combination with an encapsulated fluorescent dye, administered in the acceptor chamber of conventional PAMPA microplates. Because the detection principle relies on the molecular recognition of an analyte by the receptor and the associated fluorescence response, concentration changes of any analyte that binds to the receptor can be monitored (molecules with aromatic residues in the present example), regardless of the spectroscopic properties of the analyte itself. Moreover, because the fluorescence of the (upper) acceptor well can be read out directly by fluorescence in a microplate reader, the permeation of the drug through the planar lipid layer can be monitored in real time. Compared with the traditional assay, RT-PAMPA allows not only quantification of the permeability characteristics but also rapid differentiation between fast and slow diffusion events.

## Introduction

**Scheme 1:**
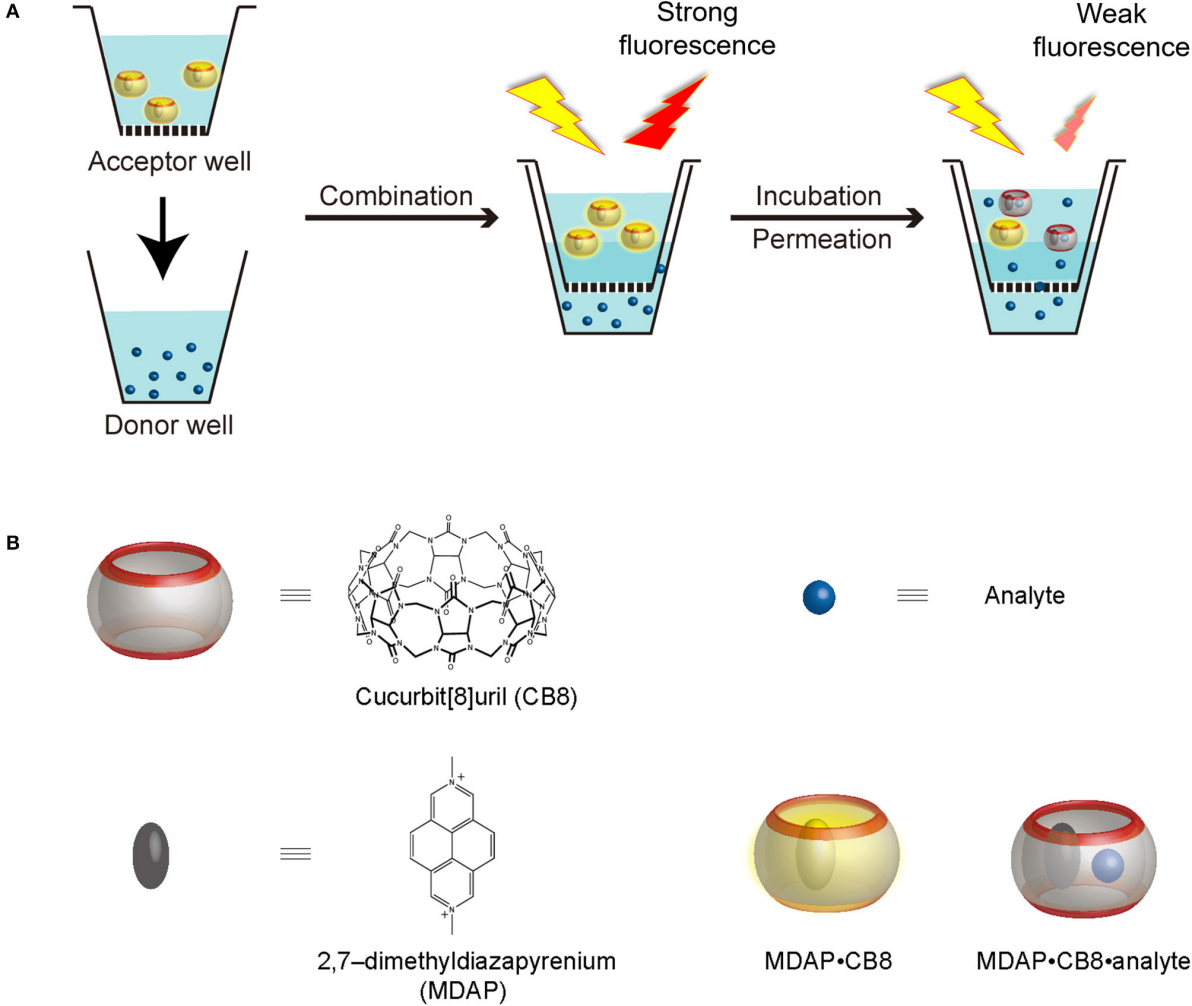
**(A)** Working principle of RT-PAMPA and **(B)** schematic representation, as well as chemical structures of CB8, MDAP (as ${\text{PF}}_{6}^{-}$ salt), the MDAP∙CB8 complex, as well as the MDAP∙CB8∙analyte ternary complex.

Drug absorption is a key factor in the evaluation of potential active pharmaceutical ingredients at the early stage of drug development. For orally administered drugs, the solubility and permeability in the gastrointestinal system determine whether the drug concentration can reach a sufficient therapeutic physiological level. The permeability of a drug is commonly estimated with empirical physicochemical chemometric methods (e.g., QSAR models) (Hou et al., 2004), cell-based assays (Caco-2 assay) (Artursson et al., 2001), or artificial membrane–based assays [e.g., parallel artificial membrane permeability assay (PAMPA)] (Kansy et al., 1998; Faller, 2008). While the cellular Caco-2 model is a highly informative screening model, there are limitations such as long differentiation period, poor interlaboratory reproducibility, and economic considerations (Hayeshi et al., 2008). PAMPA has been introduced 20 years ago as a complementary tool to evaluate the passive transportation of drugs; the assay is conducted with specially designed and commercially available “sandwich-type” multiwell microplates, which consist of an acceptor plate and a donor plate that are separated by a lipid-infused microporous filter (Scheme 1A).

PAMPA has been used in combination with, or as an alternative to the Caco-2 assay, to improve efficiency and reproducibility (Berben et al., 2018). Variables in PAMPA have been extensively studied, including the effect of stirring of the lower water layer, pH, and sink conditions. The thickness of the unstirred water layer, for example, is a critical parameter, especially for large drug molecules; in some circumstances, it can be a rate-limiting step (Wohnsland and Faller, 2001; Nielsen and Avdeef, 2004). The pH of the drug solution is another important factor, as the neutral form of a compound regularly has a larger permeability than the ionic one (Oja and Maran, 2015). Besides, the *in vivo* absorptive sink condition (e.g., a pH gradient at the two sides of the membrane, blood flow, and serum proteins) has also been considered, which led to the development of the double-sink PAMPA method, as a variant (Avdeef, 2003; Avdeef et al., 2004). Because of its implementation as a single-point assay, the permeability value with PAMPA is obtained after a specific incubation time, typically 10–16 h (Sugano et al., 2001). At this end point, the permeation process is interrupted by separating the acceptor from the donor plate and the samples of the individual wells are transferred to microplates suitable for UV-Vis quantification; for drug candidates with insufficient UV-Vis absorption, liquid chromatography–mass spectrometry (LC/MS) techniques are required for quantitative analysis, adding instrumental–analytical work for each and every well of analyte (Hanlan et al., 2003).

In this experimental study, we administer a fluorescent artificial receptor (FAR) into the acceptor well of PAMPA (Scheme 1A). The FAR is known to rapidly complex selected analytes by molecular recognition and thereby changes its fluorescence, either quenching or enhancement. Therefore, when the analyte is administered to the donor well of the PAMPA setup, its permeation through the artificial membrane connecting the acceptor and the donor plate can be followed in real time through a fluorescence intensity change, resulting in the setup of a real-time PAMPA (RT-PAMPA). From a chemical point of view, FARs are composed of a supramolecular reporter pair, that is, a macrocyclic host and a corresponding fluorescent dye molecule. Examples of commonly used FARs are 2,7-dimethyldiazapyrenium (MDAP) in combination with cucurbit[8]uril (CB8) (Sindelar et al., 2005), berberine in a pair with cucurbit[7]uril (Megyesi et al., 2008), and lucigenin together with *p*-sulfonato-calix[4]arene (Guo et al., 2011). The sensing capabilities of these artificial receptors have already been demonstrated in the context of analyte transport through membrane porins (Ghale et al., 2014; Assaf et al., 2019; Barba-Bon et al., 2019), for binding constant measurements (Florea and Nau, 2011), for enzymatic reaction monitoring (Ghale et al., 2011), and to demonstrate analyte uptake into cells (Norouzy et al., 2015). The different FARs can be further classified according to their operational principle (competitive and associative binding mechanism) (Sinn and Biedermann, 2018) or their phenomenological fluorescence intensity response upon analyte binding (“switch-on” vs. “switch-off”) (Florea et al., 2012; Ghale and Nau, 2014; Biedermann et al., 2015). Among those, the first one, MDAP∙CB8, emerged as the most broadly applicable one (see chemical structures in Scheme 1B), because it responds to a wide range of analytes with aromatic residues, which covers also numerous drugs and biomolecules of interest. The MDAP∙CB8 receptor operates through an associative mechanism (formation of a ternary complexes with the analyte, Scheme 1B), and it displays a switch-off fluorescence response, due to intracavity quenching of the dye in the ternary complex. It is noteworthy that our present technical implementation draws inspiration from a recent assay method (FAR membrane assays), which has originally been developed for liposomal solutions to monitor analyte permeation through the vesicular membranes (Biedermann et al., 2020).

To simplify the methodology in our present proof-of-concept study, the traditional PAMPA setup was employed, that is, without considering the unstirred water layers and sink conditions. As an important technical simplification of RT-PAMPA, the permeation process can be directly followed by measuring the fluorescence in the (upper) acceptor well in the microplate reader throughout the incubation period, that is, without the need to disassemble the plates as is common in the original UV-Vis and LC/MS implementations. Accordingly, the RT-PAMPA method differs not only chemically, because of the utilization of FARs, but also operationally, because of the direct fluorescence read-out. The latter is especially helpful when dealing with fast permeating drugs, as the lag time until the first read-out can be significantly reduced. It is also possible, as an additional perspective, to extract mechanistically relevant information, because the actual permeation kinetics can be measured, e.g., to assess saturation or lag effects or to differentiate monoexponential from multiexponential kinetic profiles. We found that the resulting RT-PAMPA, employing MDAP∙CB8 as FAR, responds very sensitively to electron-rich aromatic structures, such as indole. The methodology has been validated with different lipid solutions, e.g., 1,2-dioleoyl-*sn*-glycero-3-phosphocholine (DOPC), and cosolvents, and was shown to work robustly under various test conditions.

## Materials and Methods

CB8 was purchased from Strem Chemicals; the content was determined by ^1^H NMR integration. MDAP and cucurbit[7]uril were synthesized according to the literature methods (Márquez et al., 2004; Cejas and Raymo, 2005). Berberine was purchased from Sigma–Aldrich and used without further purification. DOPC was purchased from TCI and used as received. Avanti PAMPA lipid blend I was purchased from Sigma; the lipid blend was a combination of DOPC and stearic acid at a ratio of 80:20 (wt/wt) and dissolved in dodecane with 1% ethanol. Analytes were purchased from Sigma–Aldrich or TCI and used without further purification. The 96-well Multiscreen IP filter plates (MAIPNTR10) were purchased from Merck Millipore, which are based on hydrophobic polyvinylidene fluoride as filter material. The donor plate was taken from the Corning Gentest precoated PAMPA system. For the screening experiments, 96-well black polystyrene microplates from Corning were used.

### Parallel Artificial Membrane Permeability Assay

Analyte solutions were prepared at 1 mmol/L in Millipore water or cosolvents as indicated. The MDAP∙CB8 reporter pair solution contained 5 μmol/L of MDAP and 6 μmol/L of CB8 that were dissolved in Millipore water. Two lipid solutions were used, including the commercial Avanti PAMPA lipid blend I and the self-prepared 2% DOPC in dodecane. To proceed with the assay, 5 μL of the lipid solution was pipetted to the microporous filter and allowed to impregnate evenly; 300 μL of the analyte solution was transferred to the lower donor well, and 100 μL of the reporter pair solution was transferred to the upper acceptor well. The acceptor plate was combined with the donor plate after the solutions had been loaded to define the start point of the assay. Fluorescence readings were taken on a Jasco FP-8500 fluorimeter equipped with a microplate reader with an excitation wavelength at 418 nm and monitored at an emission wavelength of 449 nm.

### Screening Test

Analyte screening and solvent effect studies were carried out in 96-well microplates; 150 μL of the reporter pair solution was pipetted into each well, followed by the addition of 15 μL of the analyte solution or solvent. For analytes that were insufficiently water-soluble but that dissolved with organic cosolvents, the final organic content was 2% after mixing with the reporter pair solution. Fluorescence was measured with the same parameters as for PAMPA. Intensity changes upon addition of the analyte solutions were recorded, and solvent-induced changes (background) were subtracted.

## Results and Discussion

### Analyte Screening

**Figure 1:**
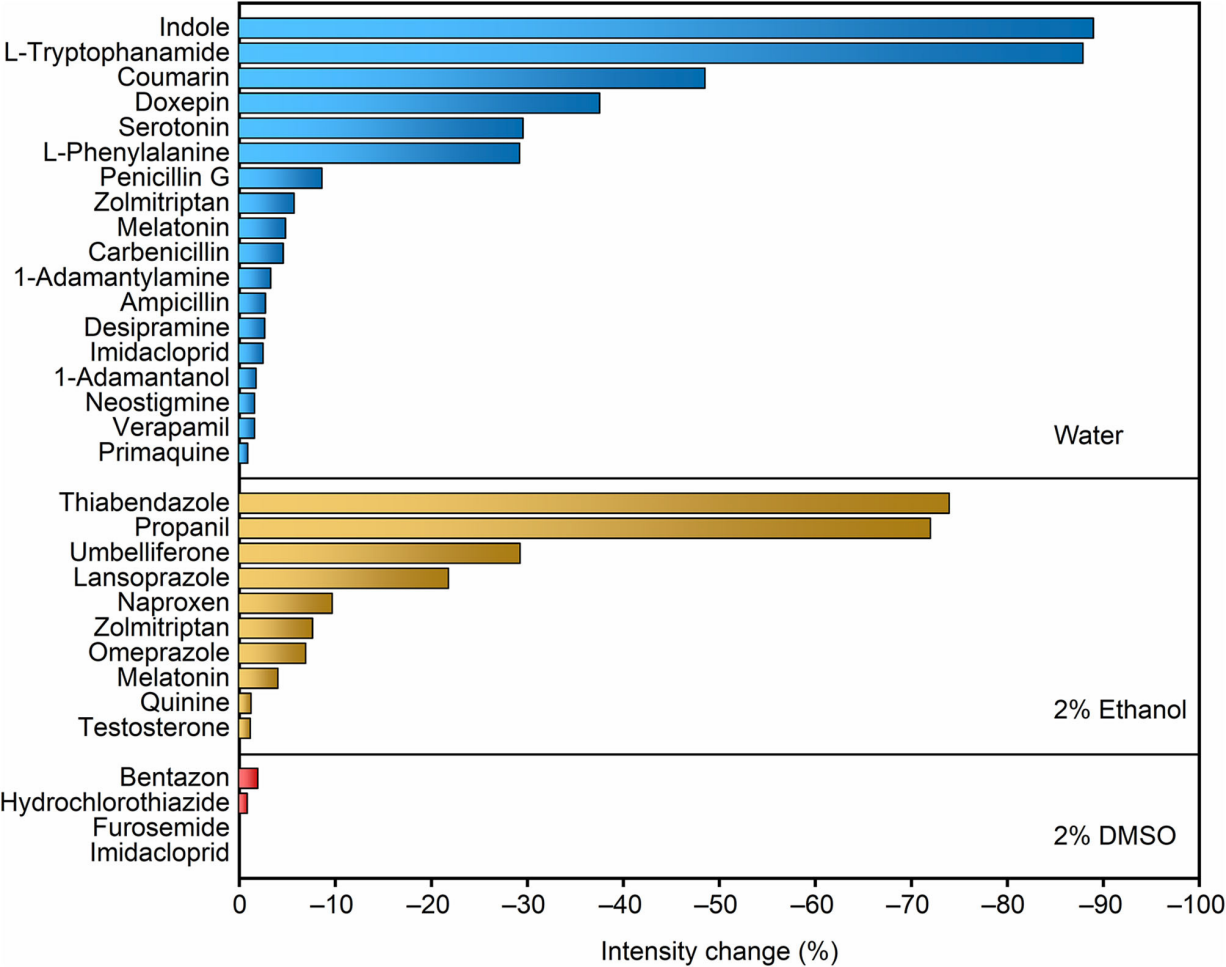
Screening of the fluorescence response of the MDAP∙CB8 receptor upon addition of different analytes dissolved in water, 2% ethanol, and 2% DMSO, from top to bottom panels. The relative fluorescence intensity decrease upon addition of 20 equiv. of the corresponding analyte to 5 μmol/L MDAP∙CB8 reporter pair solution is shown.

A recent report on the permeation of analytes across liposomal membranes tested MDAP∙CB8 and other fluorescent complexes as reporter pairs, providing us valuable information on the potential analytes that could be first implemented in the RT-PAMPA format (Biedermann et al., 2020). To establish RT-PAMPA, we started with a small-scale analyte screening procedure, in order to eliminate false-negative results caused by the lack of response to the artificial receptor. These experiments could be performed in homogeneous solution, because only the fluorescence response of the receptor toward the analyte is being determined. A set of 29 analytes prepared in aqueous solution or with organic cosolvents has been examined for their response toward MDAP∙CB8. The fluorescence intensity changes upon addition of the analytes are visualized in Figure 1. Most analytes caused a significant decrease of the fluorescence intensity, and changes >10% were considered to be sufficiently strong to be readily followed by RT-PAMPA, where a lower sensitivity was expected because of the temporal requirement of permeation. Indeed, the concentrations of the analytes in the fluorescence response experiments were conservatively set as 0.1 mmol/L, below the test concentrations used in the subsequent RT-PAMPA (1 mmol/L), such that already the permeation of a fraction of analyte from the donor to the acceptor well should ensure a fluorescence response in the upper.

The compounds that caused the largest fluorescence changes have several common structural features. MDAP possesses a positively charged conjugated organic moiety, which is predestined to get engaged in electrostatic interactions with electron-rich structures (e.g., indoles and coumarins). On the other hand, because of the size selectivity of the CB8 cavity with MDAP inside, large molecules and those with large branched residues showed no or weak associative binding (e.g., quinine, furosemide, primaquine, zolmitriptan, 1-adamantylamine, and 1-adamantanol). In general, an electron-rich, conjugated linear structure is preferred by the selected reporter pair. Naturally, because the sensing in RT-PAMPA is based on a molecular recognition process with an associated selectivity, the range of accessible analytes for a particular FAR is limited. Conversely, the technique is not limited to a single receptor. For example, berberine in combination with cucurbit[7]uril and lucigenin in a pair with *p*-sulfonato-calix[4]arene are alternative reporter pairs, which respond to different classes of analytes, e.g., adamantyl and polycyclic aliphatic derivatives for the former (Megyesi et al., 2008; Moghaddam et al., 2011) and compounds with tethered trimethylammonium groups for the latter (Arena et al., 2004; Guo et al., 2008, 2011). Through a combination of different receptors, the potential of RT-PAMPA can be expanded, although we concentrate herein on the demonstration for a single one, MDAP∙CB8.

### Influence of Cosolvent

Buffers and organic cosolvents are widely used in traditional PAMPA to increase drug solubility in water. Pharmaceutical libraries routinely involve compound storage in dimethyl sulfoxide (DMSO), and compatibility with this cosolvent always needs to be ensured. Accordingly, we examined the influence of organic cosolvents, including water mixtures with DMSO, methanol, and ethanol. These control experiments were important, because the molecular-recognition process with synthetic macrocyclic cavities, such as that in CB8 or the MDAP∙CB8 complex, is frequently driven by hydrophobic interactions, which are being modulated in different solvents.

**Figure 2:**
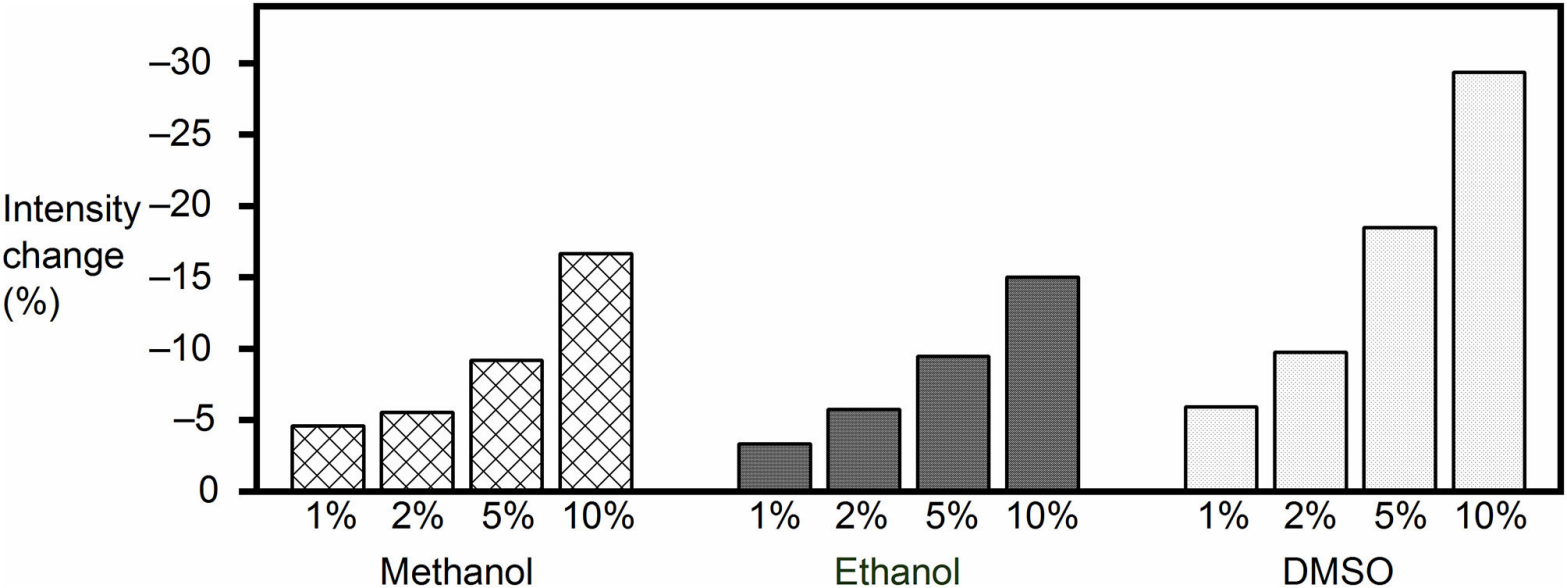
Fluorescence intensity change of the MDAP∙CB8 reporter pair solution upon addition of methanol, ethanol, or DMSO to the desired final concentration.

The final concentration of the organic proportion ranged from 1 to 10% upon direct addition into the reporter pair solutions. As expected from a leveling of the hydrophobic driving force for receptor–analyte (host–guest) complexation in organic solvents, the fluorescence intensity decreased in all cases, and the effect became significant as the organic proportion increased (Figure 2), with the largest drop (by 29%) being observed for 10% DMSO content. However, the response of the FAR was quite stable at low organic solvent proportion (1%), with the fluorescence intensity being reduced by only 5, 3, and 6% with methanol, ethanol, and DMSO, respectively. Although the results suggested that high proportions of organic cosolvent needed to be avoided, because they would reduce the sensitivity of the assay, the RT-PAMPA application should be sufficiently robust to allow common low DMSO proportions, and most importantly, the effect of organic cosolvents on the fluorescence response of the receptor was found to be predicable and systematic.

### Influence of Lipid Layer

In PAMPA, a lipid barrier needs to be crossed when an analyte moves from the donor plate into the acceptor plate. The initial PAMPA used egg lecithin as the lipid bilayer membrane (Kansy et al., 1998; Faller, 2008); other options such as hexadecane (Wohnsland and Faller, 2001) and lipid mixtures (Sugano et al., 2001) have been developed subsequently. Herein, we selected a commercial Avanti lipid solution with good fluidity in all PAMPA experiments. As an alternative common lipid, 2% DOPC in dodecane solution has been used for comparison. Before proceeding to the RT-PAMPA experiments, we monitored the stability of the FAR in the acceptor well when in contact with the donor well in the absence of added analyte. Over a period of 7-h incubation time, a slight decrease of the fluorescence was monitored, which amounted to ca. 5 ± 2% for the 2% DOPC lipid and to 12 ± 3% for the Avanti premixed lipid (see Supplementary Material). Presumably, this decrease reflects some dissolution of the receptor, or a component of it, in the lipid layer. Interestingly, this drop was independent, within error, of the presence of organic cosolvent (up to 20%) in the donor layer, which demonstrates that the diffusion of the polar organic cosolvents through the lipid does not contribute to the fluorescence changes in this time scale.

### RT-PAMPA

**Figure 3:**
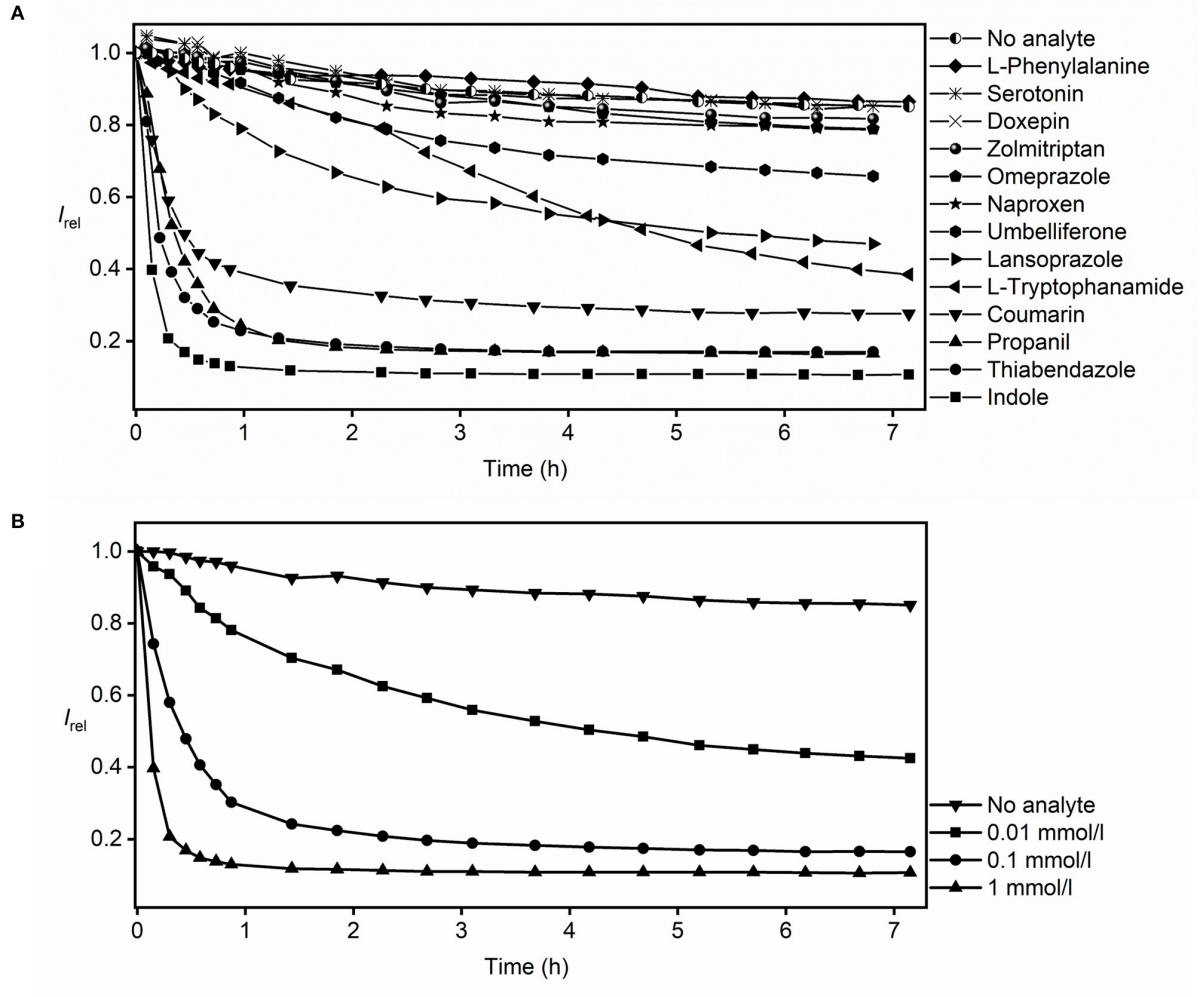
RT-PAMPA with the MDAP∙CB8 fluorescent receptor and Avanti lipid films for monitoring the permeation process of **(A)** selected analytes that show binding to the fluorescent receptor and **(B)** indole at different analyte concentrations.

In the actual RT-PAMPA experiments with MDAP∙CB8 and the Avanti lipid (Figure 3A), the fluorescence decrease recorded from the different analytes needs to be compared with the fluorescence response in the absence of analyte, where also a slight decrease is observed (see previous section). Accordingly, significant fluorescence responses, indicative of analyte permeation through the lipid layer, were observed for umbelliferone, lansoprazole, l-tryptophanamide, coumarin, propanil, thiabendazole, and indole. l-Phenylalanine, serotonin, doxepin, and zolmitriptan showed no significant fluorescence response in comparison to the absence of analyte. Because these analytes show a strong fluorescence response in homogeneous solution (Figure 1), they do not or too slowly permeate through the lipid layer in RT-PAMPA.

For compounds that show insignificant fluorescence response/binding toward the MDAP∙CB8 reporter pair, such as 1-adamantylamine or 1-adamantanol (Figure 1), other reporter pairs can be in principle employed. To demonstrate, we conducted exploratory experiments (see Supplementary Material) with the berberine∙cucurbit[7]uril reporter pair, which showed a strong fluorescence response, indicative of effective permeation, toward 1-adamantylamine and 1-adamantanol. The variability of the reporter pairs presents an additional asset of RT-PAMPA, which at the same time limits its general applicability for screening larger libraries with a single reporter pair. Among the tested reporter pairs, MDAP∙CB8 was, however, found to surpass the other candidates in terms of accessible analyte scope.

The RT-PAMPA was carried out with two kinds of lipid membranes, the commercial Avanti PAMPA blend I and the laboratory prepared 2% DOPC dodecane solution (see Supplementary Material for all results with 2% DOPC). A better resolution between the different analytes was observed with the commercial lipid layer, possibly because it is more permeable by dissolving 1% ethanol and stearic acid in dodecane besides just DOPC. The comparison also afforded differences in permeation profiles of some analytes. For example, umbelliferone, which showed an appreciable permeability through the Avanti lipid (Figure 3A), did not permeate to a significant extent through the 2% DOPC lipid under RT-PAMPA conditions. As a particular advantage of RT-PAMPA, fast and slow permeation characteristics can be immediately differentiated from the permeation curves. Fast permeation is reflected by a sharp drop of the fluorescence during the initial several minutes, e.g., for coumarin, propanil, thiabendazole, and indole, revealing that a significant amount of the analyte has rapidly crossed the membrane model. In contrast, slowly permeating compounds such as umbelliferone, lansoprazole, and l-tryptophanamide showed a fluorescence response on the time scale of several hours in RT-PAMPA. As expected, the kinetics of the permeation profiles (slow, moderate, and fast) nicely correlated with the known permeabilities of the analytes. For instance, the permeability coefficients (log*P*_e_) of indole and coumarin are reported as −4.50 and −4.55, respectively, which are considered fast (Fujikawa et al., 2007). In contrast, doxepin, whose permeability coefficient is < −6 in acidic solutions (which matches our experimental condition for using doxepin hydrochloride in the donor well), is categorized as slow permeation (Oja and Maran, 2015). Meanwhile, the performance of l-tryptophanamide is moderate to slow with a permeability coefficient of −5.56 (Fujikawa et al., 2007). It should also be noted that simple (single-parameter) empirical correlations between the permeability coefficients and other physicochemical parameters of the analytes (such as their molecular size or their lipophilicity as assessed by octanol–water partition coefficients) do not apply, which emphasizes the need to experimentally determine the permeabilities of analytes with dedicated assays, such as RT-PAMPA (Fujikawa et al., 2007; Biedermann et al., 2020).

RT-PAMPA is in principle also suitable to extract absolute permeation coefficients and rates, although these are strongly dependent on the actual experimental conditions (see above) and can only serve as a relative comparison and/or rough method-to-method comparison. The quantitative analysis requires that a plateau value for the final fluorescence intensity has been reached, which is indicative of analyte diffusion having reached equilibrium and/or complete consumption of the reporter pair. To differentiate between the two options, experiments at different analyte concentrations can be performed. For indole, see Figure 3B; these experiments (from 0.01 to 1 mmol/L) revealed variations of the plateau with varying indole concentration, which signifies that the diffusion equilibrium is actually being attained and that the artificial receptor is not being consumed; in the latter case, the same plateau would be reached with different rates. Under those conditions of equilibration and with the knowledge of the binding affinity between the analyte and the reporter pair, the absolute permeability coefficients of the analyte can be extracted (see Supplementary Material). The effective permeability coefficient of indole (log *P*_e_) was calculated as −4.57 (as an average value), which compares favorably with that reported by others (log *P*_e_ = −4.50, with 10% egg lecithin solution as the lipid barrier) (Fujikawa et al., 2007). A closer inspection of the permeability coefficients calculated for different time intervals or for different indole concentrations (see Supplementary Material) shows small systematic trends (smaller values at higher analyte concentrations), which may be related to the almost complete consumption of the receptor at higher analyte concentrations and an associated larger error. Important to note, the presence of organic cosolvents in the donor well had a minor influence on the absolute permeation kinetics. To provide a specific example, we prepared 0.1 mmol/L thiabendazole samples in both 2 and 20% ethanol, and the resulting permeation curves overlapped within the time of largest changes (initial hour, see Supplementary Material).

## Conclusions

In summary, we have developed a real-time PAMPA model, referred to as RT-PAMPA, by introducing the FAR MDAP∙CB8 into the acceptor well. In contrast to the traditional single-point PAMPA, RT-PAMPA can offer multiple data point data along the entire incubation period, and it bypasses multiple incubation steps related to detection by UV-Vis absorption or measurement with LC/MS. A rational protocol to perform the RT-PAMPA involves analyte screening as a preliminary test to evaluate the response of the fluorescent receptor to the analyte. For analytes that interact with the reporter pair, the complexation-induced fluorescence change in RT-PAMPA can be recorded in real time along during analyte incubation. Fast and slow permeation behavior can be readily recognized from the slope of the initial intensity drop. Absolute permeability coefficients of analytes can be obtained by knowledge of the binding affinity between analyte and the reporter pair. The method validation demonstrates that RT-PAMPA can be used robustly in aqueous solution and with organic cosolvents, including DMSO. Moreover, RT-PAMPA can be flexibly modified to monitor various analyte classes by changing to a suitable reporter pair. It is also possible to monitor permeation through different lipid compositions characteristic for different biological barriers.

## Data Availability Statement

The raw data supporting the conclusions of this article will be made available by the authors, without undue reservation.

## Author Contributions

SH and WN designed the project and experiments. SH and AZ performed all PAMPA and screening experiments. AB-B performed the synthesis of MDAP. AH instructed preliminary experiments with valuable findings. SH and WN wrote the manuscript. All authors contributed to the article and approved the submitted version.

## Conflict of Interest

The authors declare that the research was conducted in the absence of any commercial or financial relationships that could be construed as a potential conflict of interest. The handling editor declared a past collaboration with the authors AH and WN.
