# Supplementary material for: Real-Time Parallel Artificial Membrane Permeability Assay Based on Supramolecular Fluorescent Artificial Receptors
Source: Front Chem. 2020 Nov 3;8:597927. doi: 10.3389/fchem.2020.597927 (PMC7673371; doi:10.3389/fchem.2020.597927)
Supplement: Supplementary file 1 [file Data_Sheet_1.PDF]

Supplementary Material

Table of Contents

1. General information
2. Blank control experiments
3. Summary of RT-PAMPA results
4. RT-PAMPA with BE•CB7 as FAR
5. Calculation of permeability

1 General information

RT-PAMPA and fluorescence titration experiments were conducted on a Jasco FP-8500 fluorimeter with an attached microplate reader. For RT-PAMPA, the analytes were prepared at 1 mmol/l, while the FARs, including MDAP•CB8 and berberine•cucurbit[7]uril (BE•CB7), were prepared at 5 μmol/l. In the first step, 5 μl of the lipid solution (Avanti PAMPA Blend I or 2% DOPC) was loaded on the microporous bottom of the acceptor well and allowed to disperse evenly. Subsequently, 100 μl of the FAR solution was loaded in the acceptor well, and 300 μl of the analyte solution was loaded into the donor well. Immediately after the solutions were loaded, the two plates were combined with the acceptor plate on top and the donor plate at the bottom. Fluorescence intensity was recorded at 449 nm with an excitation wavelength of 418 nm for the MDAP•CB8 experiments and recorded at 500 nm with an excitation wavelength of 342 nm for the BE•CB7 experiments. For the titration experiments, 2.5 ml of the 1 μmol/l MDAP•CB8 solution was placed into a 1-cm length quartz cuvette, followed by gradual addition of the indole solution. The solution was excited at 418 nm and the fluorescence spectra were recorded upon each addition of the indole solution. The fluorescence decrease at 449 nm was fitted with a 1:l binding model in order to obtain the binding constants of the MDAP•CB8•indole ternary complexes.

2 Blank control experiments

a) DMSO/water co-solvent

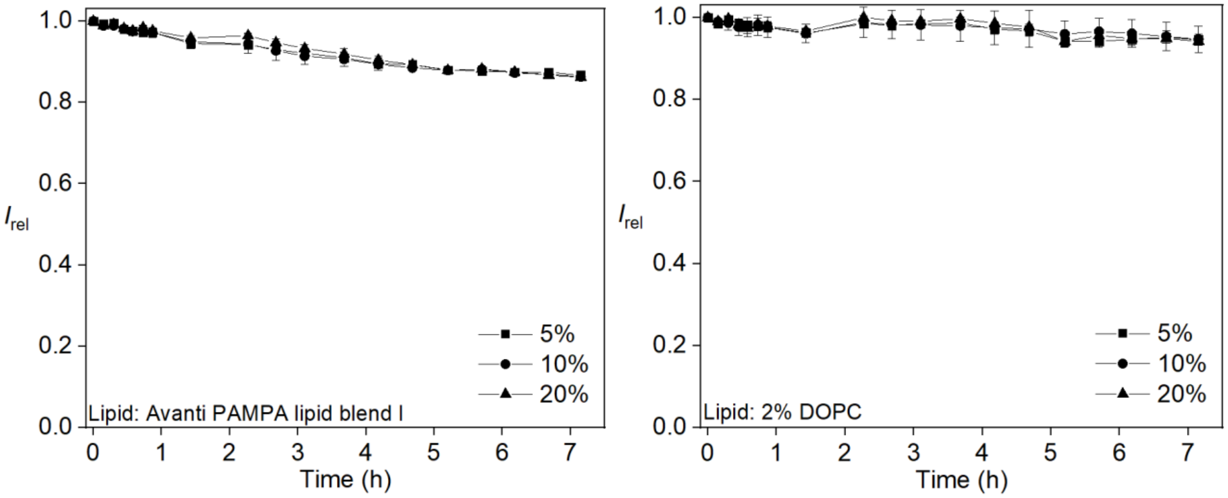

b) Ethanol/water co-solvent

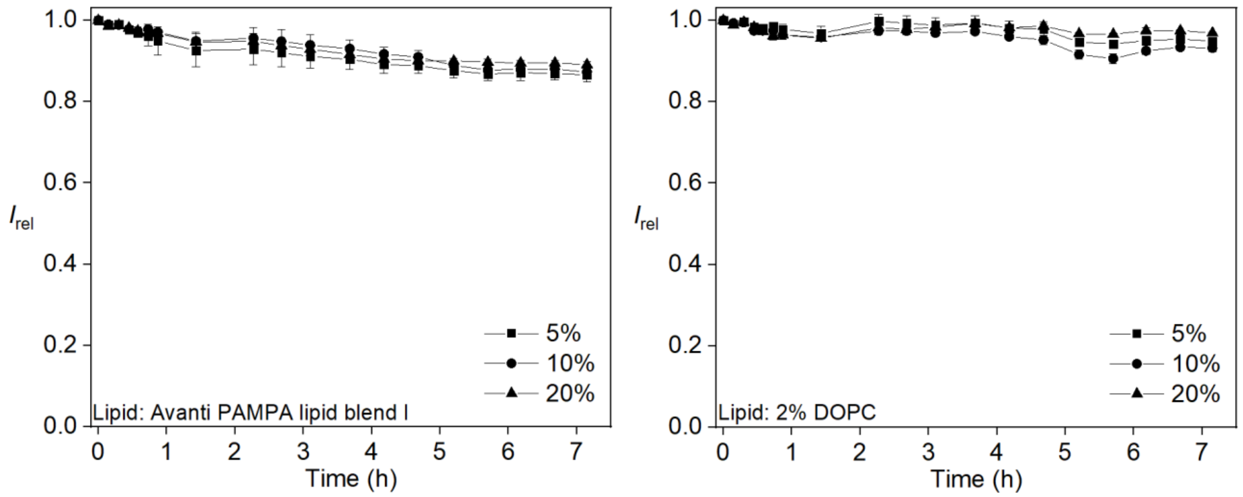

c) Methanol/water co-solvent

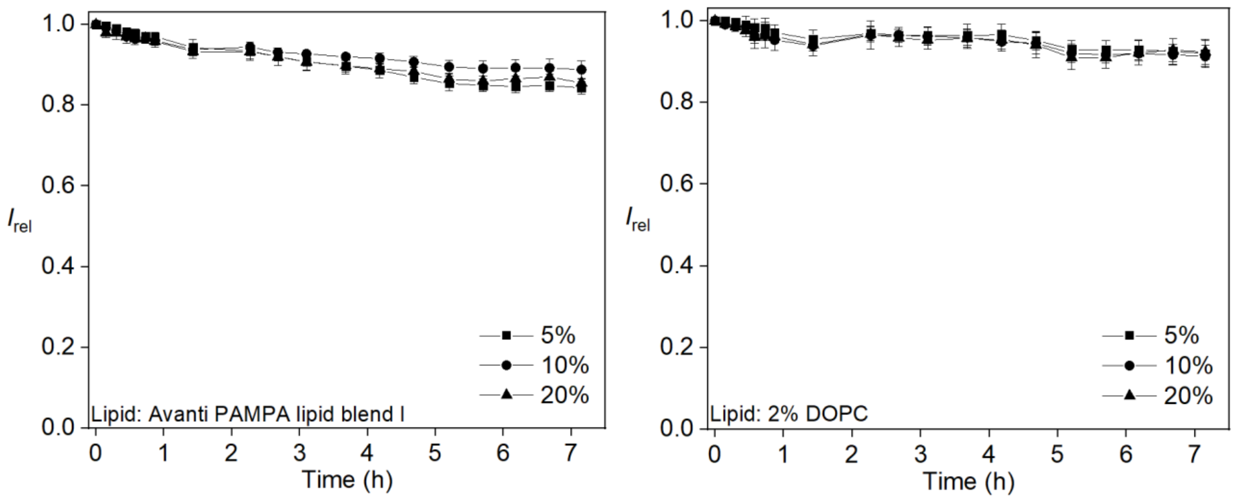

Supplementary Figure 1. Blank control results of RT-PAMPA with three organic/water co-solvents. Different content (from 5% to 20%, as indicated in the graphs) of a) DMSO, b) ethanol, or c) methanol, mixed with water, were loaded in the donor wells in the absence of analyte. Two lipid layers, including Avanti PAMPA lipid blend I (left) and laboratory prepared 2% DOPC (right) were used. Error bars show the standard deviation of three parallel experiments.

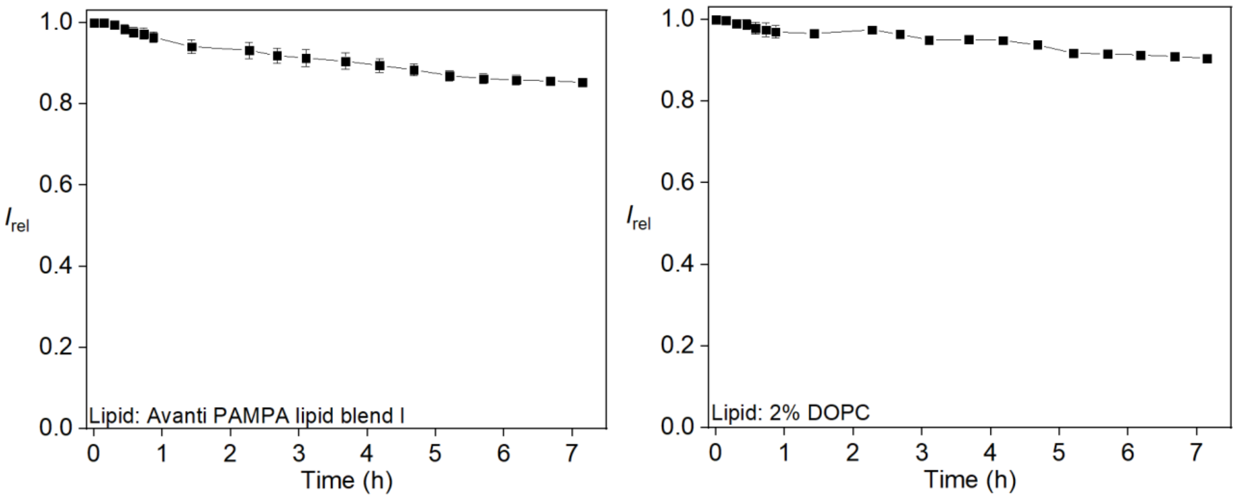

Supplementary Figure 2. Blank control results of RT-PAMPA with water. Avanti PAMPA lipid blend I (left) and laboratory prepared 2% DOPC (right) were used as lipid layers. Error bars show the standard deviation of three parallel experiments.

3 Summary of RT-PAMPA results

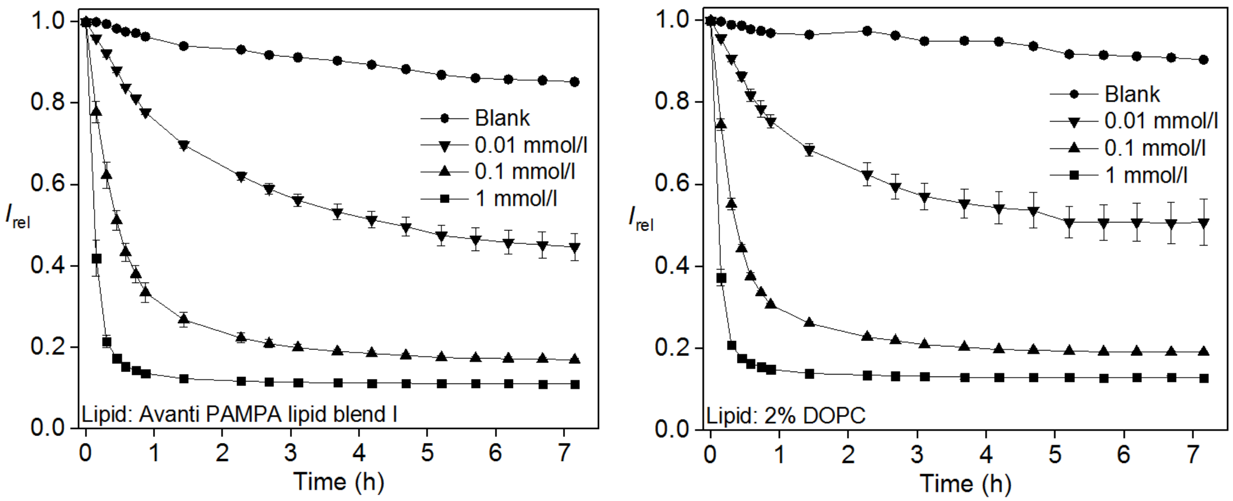

Supplementary Figure 3. Permeation curves of indole$_{(aq)}$ of different concentrations, as indicated in the graphs, through two kinds of lipid layers: Avanti PAMPA lipid blend I (left) and laboratory prepared 2% DOPC (right). Error bars show the standard deviation of three parallel experiments.

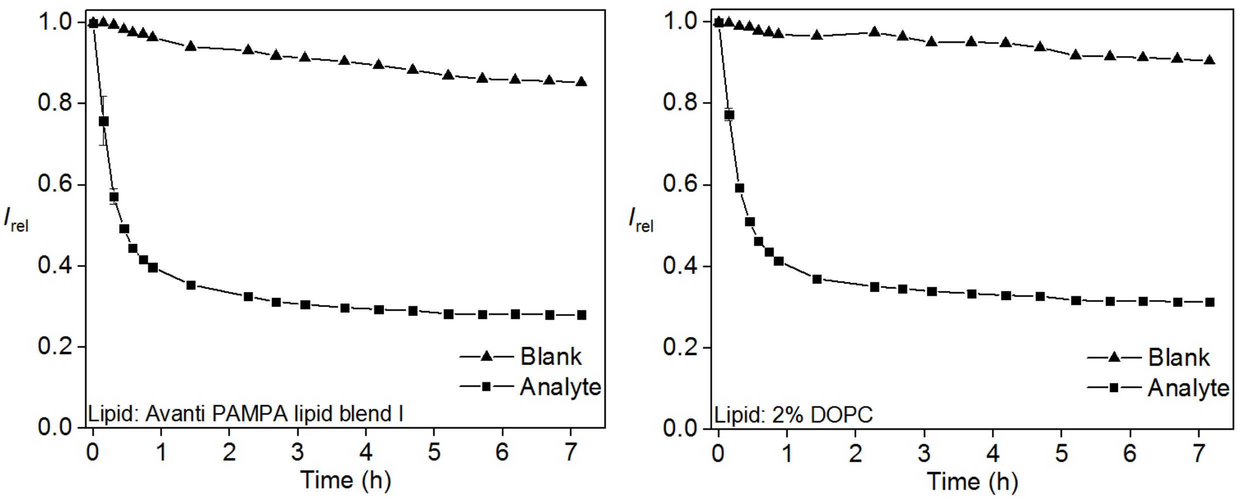

Supplementary Figure 4. Permeation curves of coumarin$_{(aq)}$ (1 mmol/l) through two kinds of lipid layers: Avanti PAMPA lipid blend I (left) and laboratory prepared 2% DOPC (right). Error bars show the standard deviation of three parallel experiments.

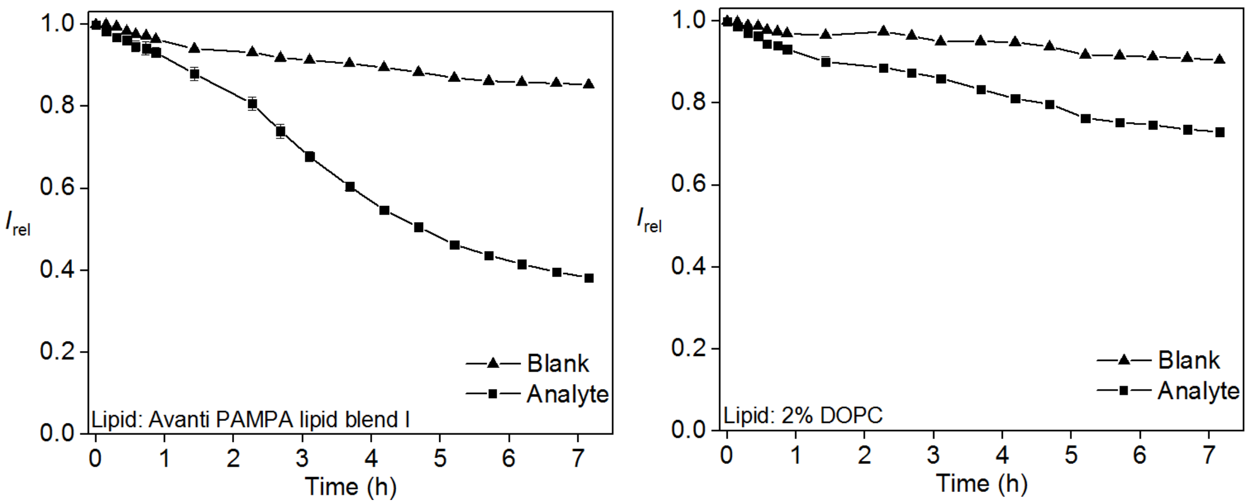

Supplementary Figure 5. Permeation curves of L-tryptophanamide$_{(aq)}$ (1 mmol/l) through two kinds of lipid layers: Avanti PAMPA lipid blend I (left) and laboratory prepared 2% DOPC (right). Error bars show the standard deviation of three parallel experiments.

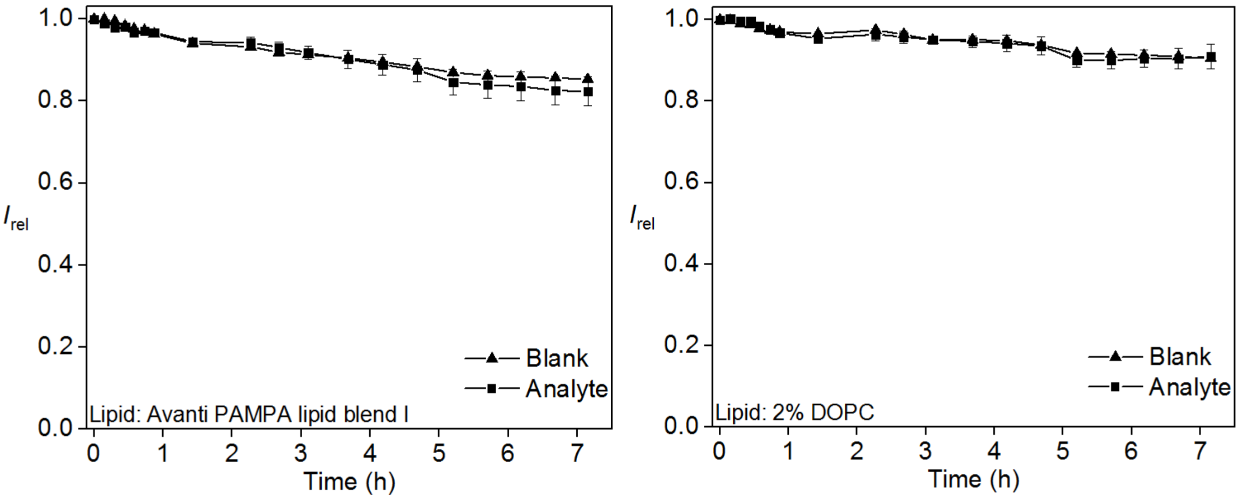

Supplementary Figure 6. Permeation curves of L-phenylalanine$_{(aq)}$ (1 mmol/l) through two kinds of lipid layers: Avanti PAMPA lipid blend I (left) and laboratory prepared 2% DOPC (right). Error bars show the standard deviation of three parallel experiments.

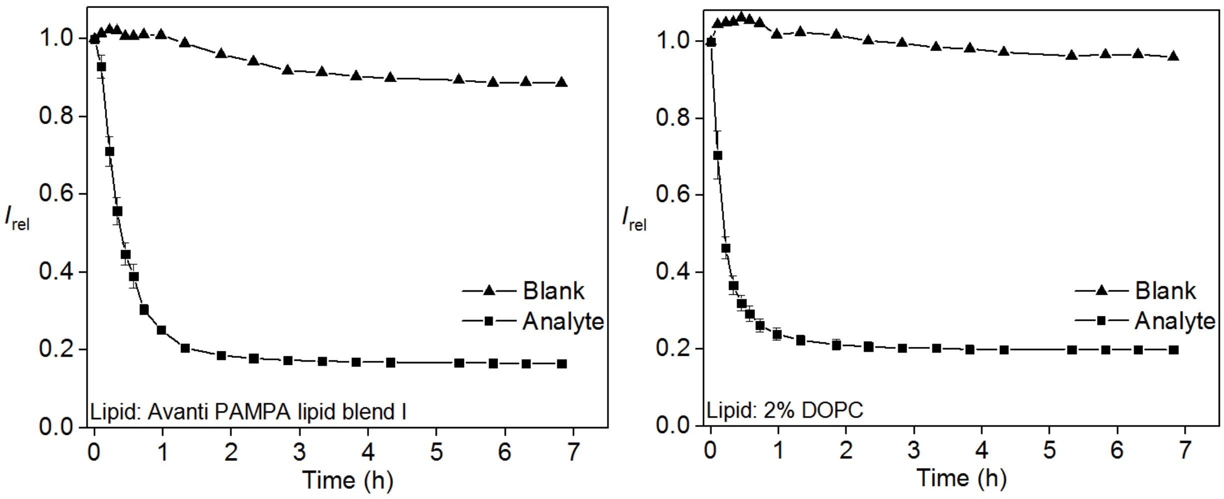

Supplementary Figure 7. Permeation curves of propanil (1 mmol/l) in 20% ethanol through two kinds of lipid layers: Avanti PAMPA lipid blend I (left) and laboratory prepared 2% DOPC (right). Error bars show the standard deviation of three parallel experiments.

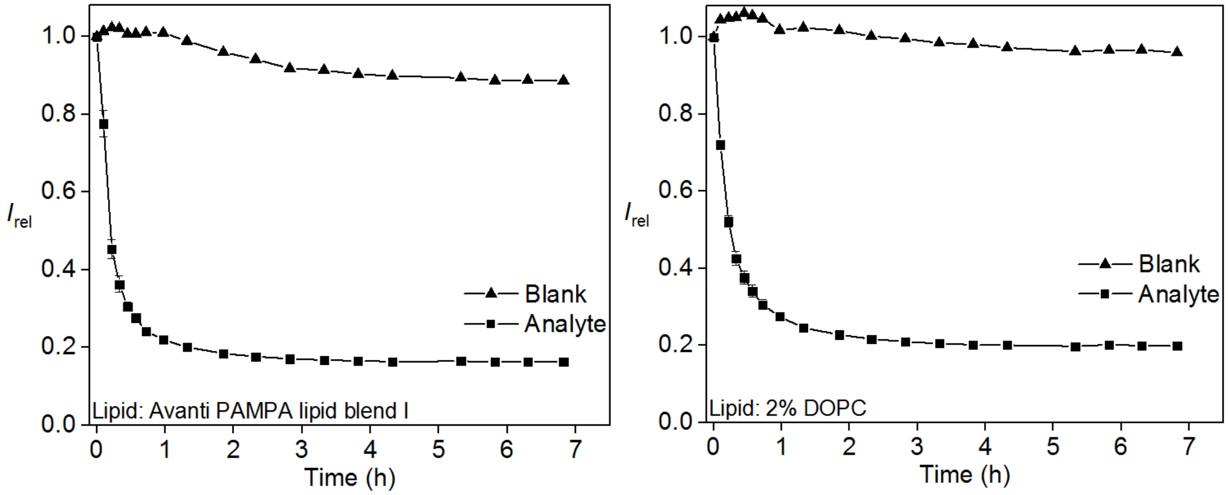

Supplementary Figure 8. Permeation curves of thiabendazole (1 mmol/l) in 20% ethanol through two kinds of lipid layers: Avanti PAMPA lipid blend I (left) and laboratory prepared 2% DOPC (right). Error bars show the standard deviation of three parallel experiments.

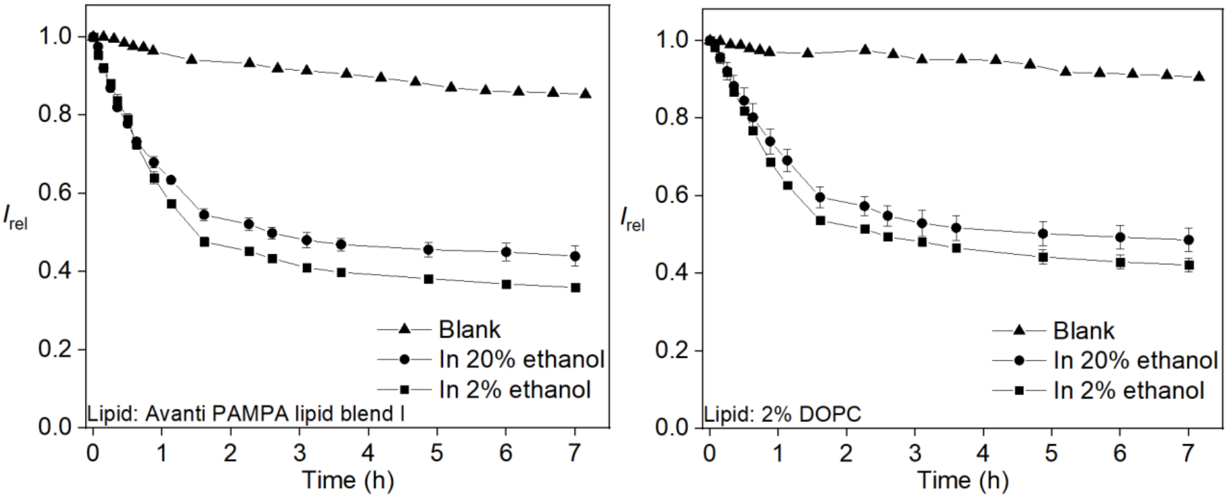

Supplementary Figure 9. Permeation curves of thiabendazole (0.1 mmol/l) in 2% and 20% ethanol, respectively, through Avanti PAMPA lipid blend I (left) and laboratory prepared 2% DOPC (right). Water blanks with respective lipid barriers are compared in the graphs. Error bars show the standard deviation of three parallel experiments.

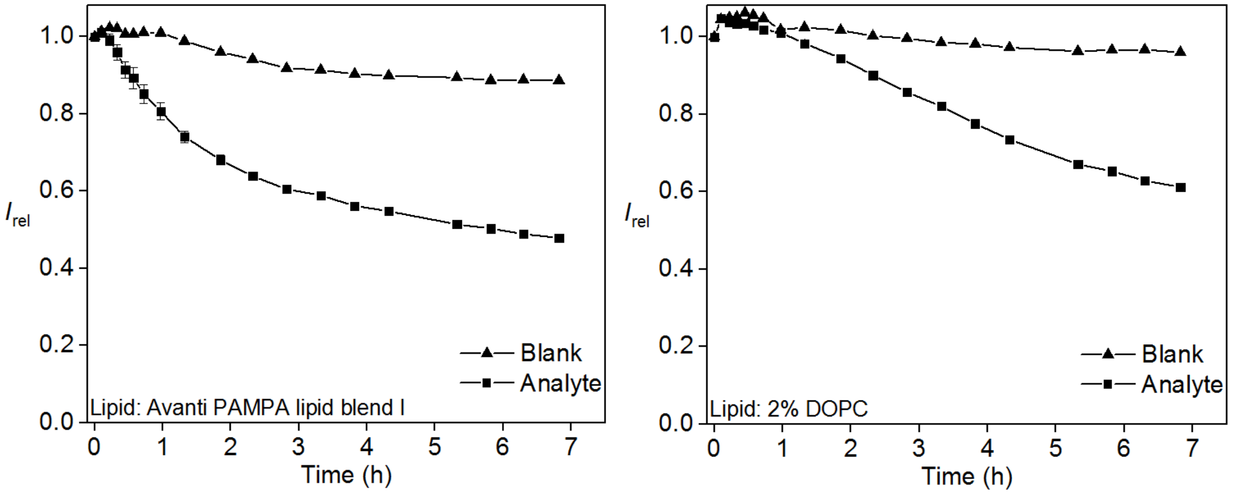

Supplementary Figure 10. Permeation curves of lansoprazole (1 mmol/l) in 20% ethanol through two kinds of lipid layers: Avanti PAMPA lipid blend I (left) and laboratory prepared 2% DOPC (right). Error bars show the standard deviation of three parallel experiments.

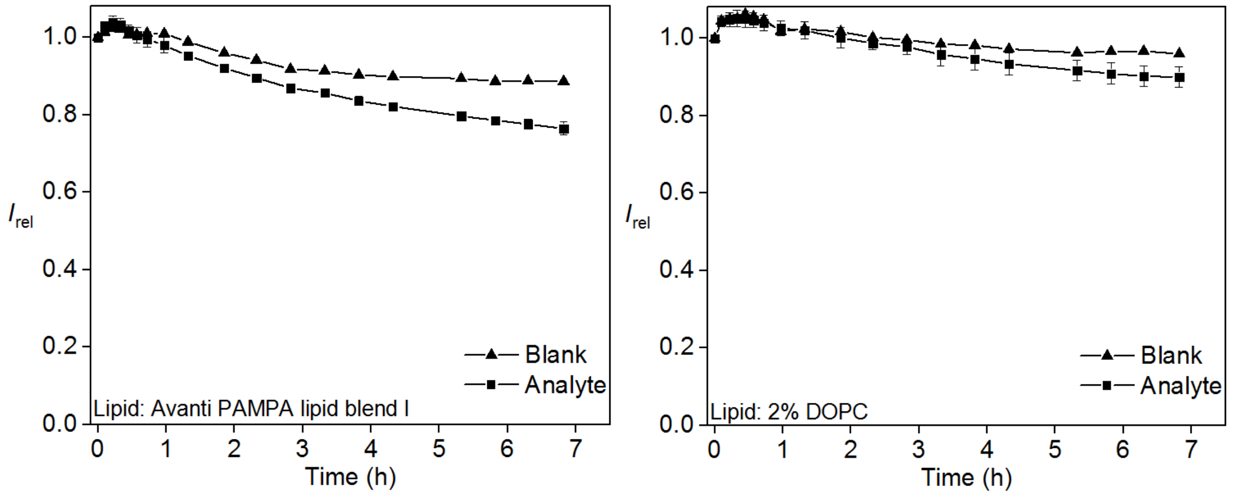

Supplementary Figure 11. Permeation curves of omeprazole (1 mmol/l) in 20% ethanol through two kinds of lipid layers: Avanti PAMPA lipid blend I (left) and laboratory prepared 2% DOPC (right). Error bars show the standard deviation of three parallel experiments.

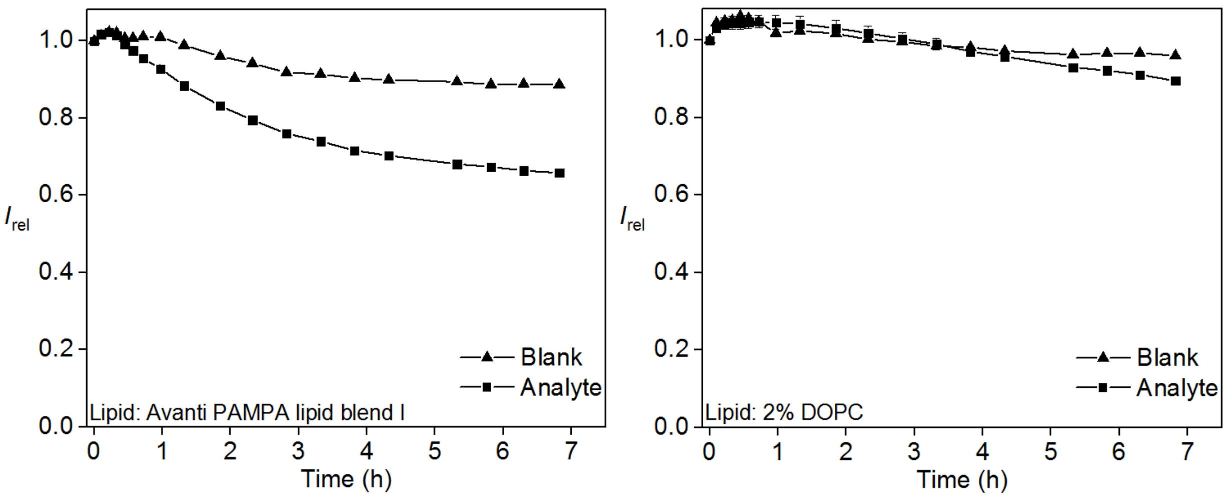

Supplementary Figure 12. Permeation curves of umbelliferone (1 mmol/l) in 20% ethanol through two kinds of lipid layers: Avanti PAMPA lipid blend I (left) and laboratory prepared 2% DOPC (right). Error bars show the standard deviation of three parallel experiments.

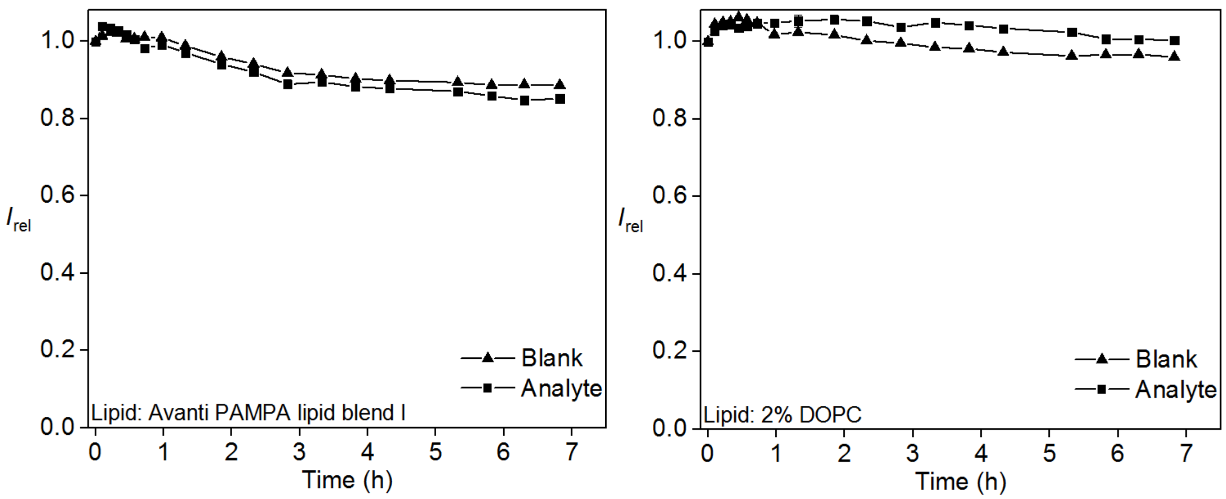

Supplementary Figure 13. Permeation curves of serotonin (1 mmol/l) in 20% ethanol through two kinds of lipid layers: Avanti PAMPA lipid blend I (left) and laboratory prepared 2% DOPC (right). Error bars show the standard deviation of three parallel experiments.

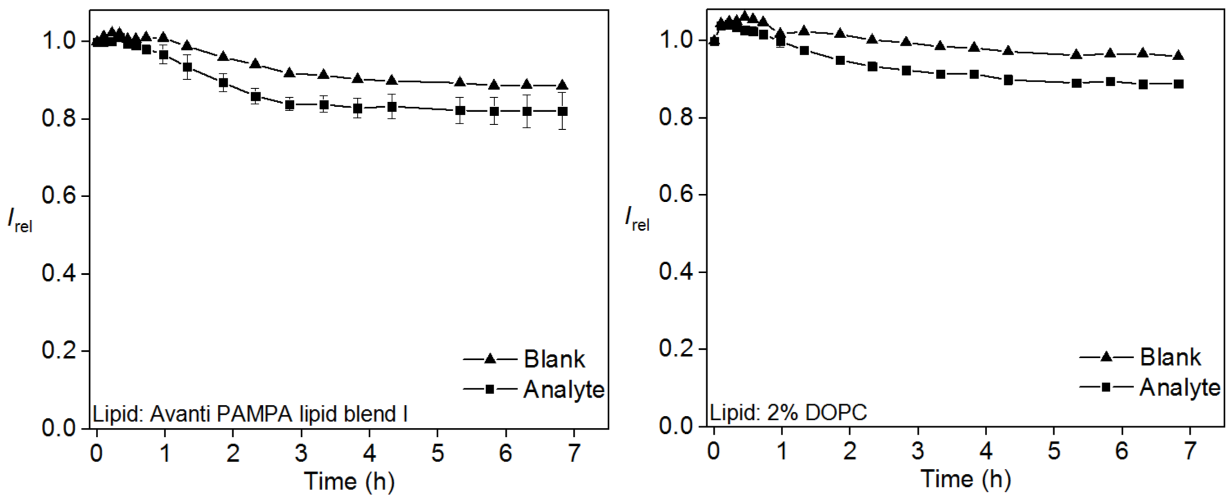

Supplementary Figure 14. Permeation curves of naproxen (1 mmol/l) in 20% ethanol through two kinds of lipid layers: Avanti PAMPA lipid blend I (left) and laboratory prepared 2% DOPC (right). Error bars show the standard deviation of three parallel experiments.

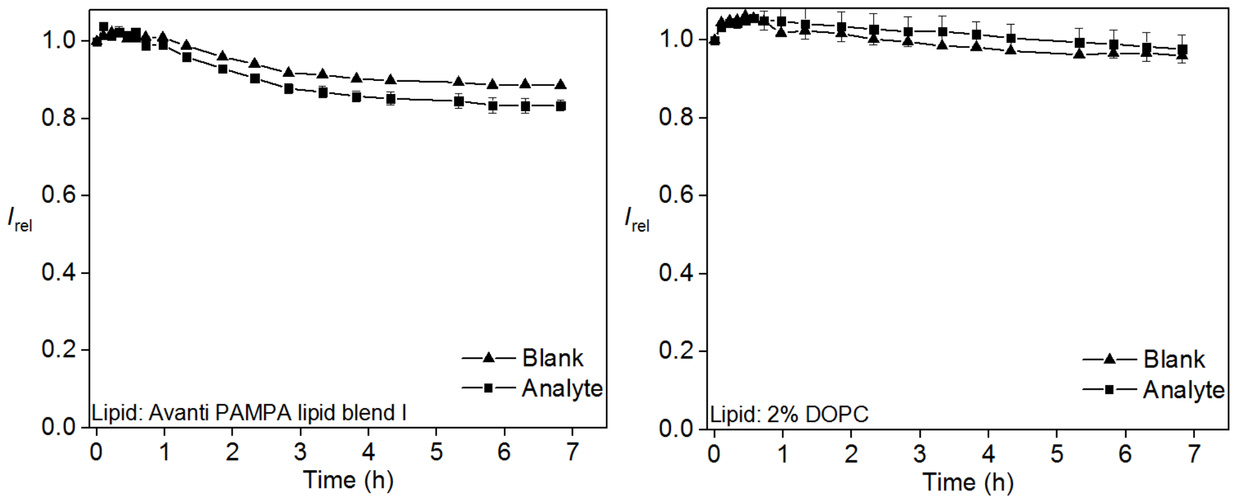

Supplementary Figure 15. Permeation curves of doxepin (1 mmol/l) in 20% ethanol through two kinds of lipid layers: Avanti PAMPA lipid blend I (left) and laboratory prepared 2% DOPC (right). Error bars show the standard deviation of three parallel experiments.

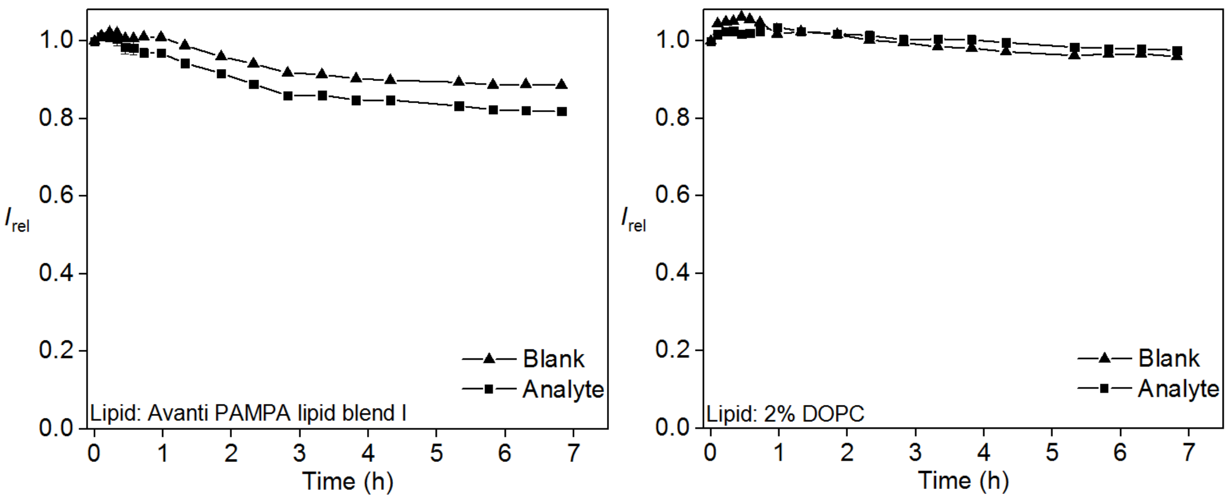

Supplementary Figure 16. Permeation curves of zolmitriptan (1 mmol/l) in 20% ethanol through two kinds of lipid layers: Avanti PAMPA lipid blend I (left) and laboratory prepared 2% DOPC (right). Error bars show the standard deviation of three parallel experiments.

4 RT-PAMPA with BE•CB7 as FAR

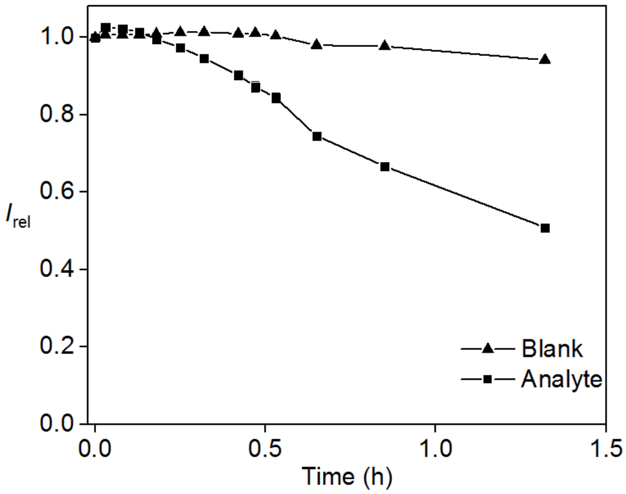

Supplementary Figure 17. Permeation curve of 10 µmol/l 1-adamantylamine in water through an Avanti PAMPA lipid layer. Error bars show the standard deviation of three parallel experiments.

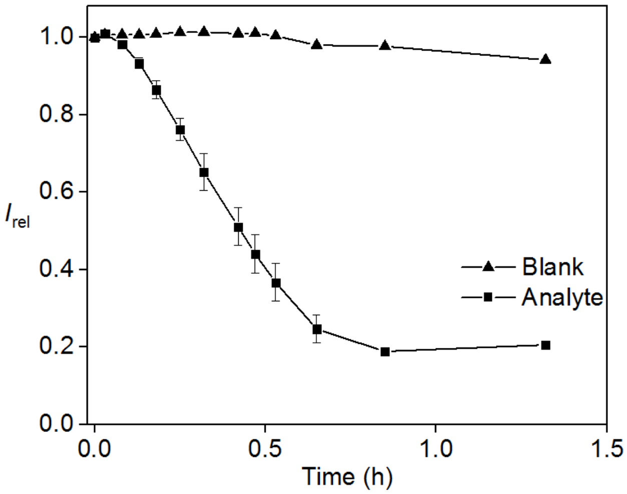

Supplementary Figure 18. Permeation curve of 10 µmol/l 1-adamantanol in water through Avanti PAMPA lipid layer. Error bars show the standard deviation of three parallel experiments.

5 Calculation of permeability

The effective permeability (P_e) of an analyte was calculated with the below equation (Wohnsland and Faller, 2001; Sugano et al., 2001):

$$logP_e = log\left\{-C \times ln\left(1 - \frac{[analyte]_{acceptor}}{[analyte]_{equilibrium}}\right)\right\}$$

in which,

$$C = \frac{V_D \times V_A}{(V_D + V_A) \times Area \times Time}$$

Based on the principle of the RT-PAMPA, the concentration of analyte in the acceptor well ($[analyte]_{acceptor}$) and the concentration at equilibrium ($[analyte]_{equilibrium}$) were obtained with the below equations, with the knowledge of the fluorescence intensity of the acceptor well and the binding constant of the ternary MDAP•CB8•analyte complex. It should be noted that the retention of analyte in the lipid layer was not taken into account.

$$[analyte]_{acceptor} = [analyte]_{complex} + [analyte]_{free}$$

$$= \frac{I_A - I_G}{I_{GA} - I_G} \times [G_0] + \frac{I_A - I_G}{K_a \times (I_{GA} - I_A)}$$

$$[analyte]_{equilibrium} = \frac{I_A - I_G}{I_{GA} - I_G} \times [G_0] + \frac{[A_0] \times V_D - (\frac{I_A - I_G}{I_{GA} - I_G} \times [G_0]) \times V_A}{V_A + V_D}$$

I_A: fluorescence intensity of the acceptor well at the chosen time point;
I_G: initial fluorescence intensity of the acceptor well;
I_{GA}: fluorescence intensity of the ternary complex;
$[G_0]$: initial concentration of FAR in the acceptor well;
$[A_0]$: initial concentration of analyte in the donor well;
V_A: volume of the solution in the acceptor well;
V_D: volume of the solution in the donor well;
K_a: binding constant of the ternary MDAP•CB8•analyte complex.

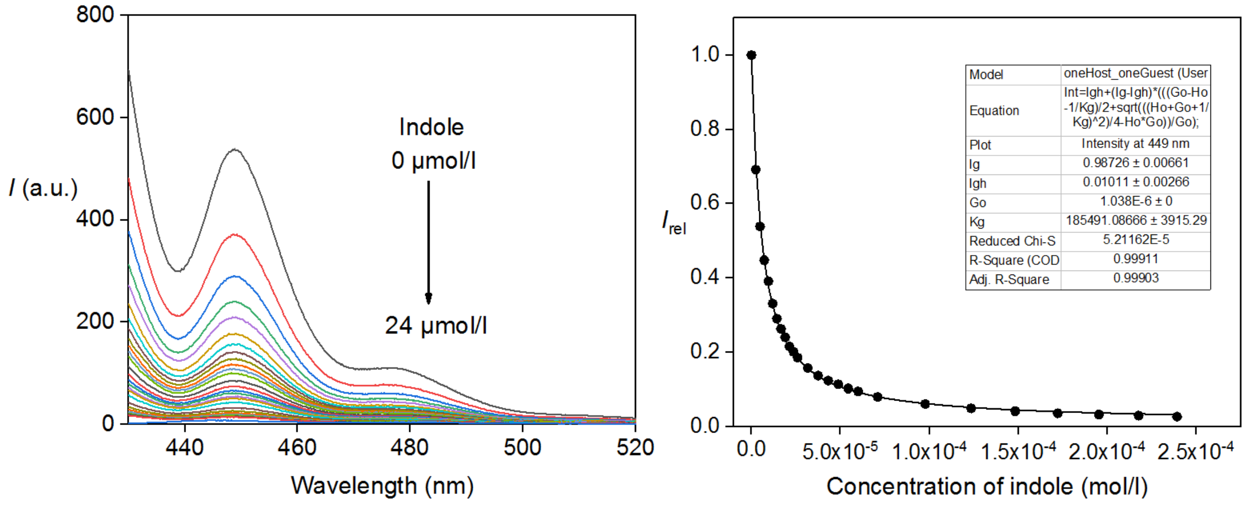

Supplementary Figure 19. Fluorescence spectral titration (left) upon addition of indole to a solution that contained 1 µmol/l MDAP•CB8 (1:1) as fluorescent artificial receptor (FAR), excited at 418 nm. The resulting fit of the fluorescence decrease (monitored at 449 nm) with indole concentration according to 1:1 binding model is shown on the right. The binding constant of the MDAP•CB8•indole ternary complex was found to be (1.9±0.1) × 10^5 M^{-1}.

Supplementary Table 1. Effective permeability coefficients (log P_e) obtained from the permeation curves of different indole concentrations (0.010-1.0 mmol/l) and at different time points.

Time (h)	log P_e		
	0.010 mmol/l	0.1 mmol/l	1.0 mmol/l
0.15	–4.55	–4.70	–5.15
0.30	–4.67	–4.72	–5.09
0.45	–4.59	–4.74	–5.17
0.58	–4.52	–4.73	–5.21
0.73	–4.53	–4.74	–5.28
0.87	–4.52	–4.72	–5.32
1.43	–4.55	–4.81	–5.49
1.85	–4.59	–4.87	–5.59

References

Wohnsland, F. and Faller, B., (2001). High-throughput permeability pH profile and high-throughput alkane/water log *P* with artificial membranes. *J. Med. Chem.* 44, 923–930. doi: 10.1021/jm001020e

Sugano, K., Hamada, H., Machida, M., and Ushio, H., (2001). High throughput prediction of oral absorption: improvement of the composition of the lipid solution used in parallel artificial membrane permeation assay. *J. Biomol. Screen.* 6, 189–196. doi: 10.1177/108705710100600309
